# Calcium-Prolactin Secretion Coupling in Rat Pituitary Lactotrophs Is Controlled by PI4-Kinase Alpha

**DOI:** 10.3389/fendo.2021.790441

**Published:** 2021-12-30

**Authors:** Marek Kučka, Arturo E. Gonzalez-Iglesias, Melanija Tomić, Rafael M. Prévide, Kosara Smiljanic, Srdjan J. Sokanovic, Patrick A. Fletcher, Arthur Sherman, Tamas Balla, Stanko S. Stojilkovic

**Affiliations:** ^1^ Section on Cellular Signaling, The Eunice Kennedy Shriver National Institute of Child Health and Human Development, National Institutes of Health, Bethesda, MD, United States; ^2^ Laboratory of Biological Modeling, National Institute of Diabetes, Digestive and Kidney Diseases, National Institutes of Health (NIH), Bethesda, MD, United States; ^3^ Section on Molecular Signal Transduction, The Eunice Kennedy Shriver National Institute of Child Health and Human Development, National Institutes of Health, Bethesda, MD, United States

**Keywords:** pituitary, lactotrophs, prolactin, regulated exocytosis, PI4-kinases, phosphoinositides, intracellular calcium

## Abstract

The role of calcium, but not of other intracellular signaling molecules, in the release of pituitary hormones by exocytosis is well established. Here, we analyzed the contribution of phosphatidylinositol kinases (PIKs) to calcium-driven prolactin (PRL) release in pituitary lactotrophs: PI4Ks - which control PI4P production, PIP5Ks - which synthesize PI(4, 5)P2 by phosphorylating the D-5 position of the inositol ring of PI4P, and PI3KCs – which phosphorylate PI(4, 5)P_2_ to generate PI(3, 4, 5)P_3_. We used common and PIK-specific inhibitors to evaluate the strength of calcium-secretion coupling in rat lactotrophs. Gene expression was analyzed by single-cell RNA sequencing and qRT-PCR analysis; intracellular and released hormones were assessed by radioimmunoassay and ELISA; and single-cell calcium signaling was recorded by Fura 2 imaging. Single-cell RNA sequencing revealed the expression of *Pi4ka, Pi4kb, Pi4k2a, Pi4k2b, Pip5k1a, Pip5k1c, *and* Pik3ca*, as well as *Pikfyve* and *Pip4k2c*, in lactotrophs. Wortmannin, a PI3K and PI4K inhibitor, but not LY294002, a PI3K inhibitor, blocked spontaneous action potential driven PRL release with a half-time of ~20 min when applied in 10 µM concentration, leading to accumulation of intracellular PRL content. Wortmannin also inhibited increase in PRL release by high potassium, the calcium channel agonist Bay K8644, and calcium mobilizing thyrotropin-releasing hormone without affecting accompanying calcium signaling. GSK-A1, a specific inhibitor of PI4KA, also inhibited calcium-driven PRL secretion without affecting calcium signaling and *Prl* expression. In contrast, PIK93, a specific inhibitor of PI4KB, and ISA2011B and UNC3230, specific inhibitors of PIP5K1A and PIP5K1C, respectively, did not affect PRL release. These experiments revealed a key role of PI4KA in calcium-secretion coupling in pituitary lactotrophs downstream of voltage-gated and PI(4, 5)P2-dependent calcium signaling.

## Introduction

Elevation in cytoplasmic calcium concentrations ([Ca^2+^]_i_) is the primary intracellular signal that controls the fusion of secretory vesicles with the plasma membrane to release hormones and neurotransmitters. This process, called regulated exocytosis, is also controlled by a complex protein system, which is preserved in organisms ranging from yeast to mammals. These proteins participate in docking, ATP-dependent priming, and fusion of secretory vesicle membranes *via* molecular interactions that have not yet been fully characterized (1, 2). Both calcium influx and calcium mobilization pathways can trigger the secretion of hormones and neurotransmitters, and the strength of the calcium-secretion coupling depends on local and global [Ca^2+^]_i_, as determined by the proximity of the secretory vesicles to calcium influx and/or intracellular calcium release channels (3).

Phosphoinositides are low abundance cellular membrane lipids that can influence regulated exocytosis (4, 5). They are generated by phosphorylation on the inositol headgroup of phosphatidylinositol (PI), to yield PI4P, PI(4, 5)P2, and PI(3, 4, 5)P3. PI4P is produced by four different PI4-kinases (PI4KA, PI4KB, PI4K2A, and PI4K2B), and each of these enzymes is associated with a different aspect of cell physiology (6). Three PI4P 5-kinases (PIP5K1A, PIP5K1B, PIP5K1C) account for the conversion of PI4P into PI(4, 5)P2 (7). An additional route for the production of PI(4, 5)P2 includes the phosphorylation of PI5P by PI5P4-kinases (8, 9). PI(4, 5)P2 is well known to contribute to calcium-secretion coupling as being the substrate for receptor activated phospholipase C to yield inositol 1,4,5 trisphosphate (IP3) and thereby controlling calcium mobilization. PI(4, 5)P2 also directly modulates ion channels and transporters and the accompanying calcium influx (10), and augments calcium-secretion coupling downstream of calcium signaling (11, 12). Class I PI3 kinases (PI3KCA, PI3KCB, PI3KCG, and PI3KCD) may also influence the level of PI(4, 5)P2 in the plasma membrane, using it as a substrate for the synthesis of PI(3, 4, 5)P3 (13), which has been implicated in calcium-secretion coupling as well (14).

Endocrine pituitary cells secrete hormones using both calcium signaling pathways: spontaneous voltage-gated calcium influx and receptor-controlled IP3-dependent calcium mobilization coupled with facilitated voltage-gated calcium influx (15). Pituitary lactotrophs, which are the subject of the present study, exhibit spontaneous plateau-bursting of electrical activity resulting in calcium transients that have sufficient amplitude to trigger basal prolactin (PRL) secretion (16). In these cells, the physiological mechanism for control of PRL release is *via* dopamine suppression of basal secretion (17). Furthermore, thyrotropin-releasing hormone (TRH) triggers IP3-dependent calcium mobilization from intracellular stores and induces additional hormone secretion (18). However, the contribution of phosphoinositides beyond the role of PI(4, 5)P2 supporting IP3 production in pituitary cell exocytosis remains poorly understood. This reflects the difficulties to up- or down-regulate the expression of enzymes that control phosphoinositide levels in cultured pituitary cells, due to resistance of these cells to conventional transfection procedures. The use of immortalized pituitary cells, such as GH3 lacto-somatotrophs, is also limited, because these cells predominantly secrete hormones in a constitutive manner.

This prompted us to use a pharmacological approach in this initial study of the role of phosphoinositides in calcium-secretion coupling in cultured pituitary lactotrophs, focusing on PI4P, PI(4, 5)P2, and PI(3, 4, 5)P3. We used wortmannin (Wm), a common PI3K inhibitor, which is effective against Class I PI3Ks in the low nanomolar concentration range (19), and against PI4KA and PI4KB in the low to high micromolar concentration range (20). Another inhibitor of a different chemical class, LY294002, also inhibits PI3K activity (21, 22). PIK93 used below 1 µM inhibits PI4KB but not PI4KA (23), while several other compounds, including GSK-A1, are potent and selective PI4KA inhibitors developed by GSK (Glaxo-Smith-Kline) using quinazolinone 28 as the starting compound (24) and characterized by Bojjireddy et al. (25). We also used IS2011B and UNC3230, reported inhibitors of PIP5K1A (26) and PIP5K1C (27), respectively. Our results revealed that inhibition of PI4Ks, but not other kinases, block PRL release downstream of calcium signaling. Additional experiments with PI4K-specific inhibitors, revealed that PI4KA is a key enzyme in establishing calcium-secretion coupling in pituitary lactotrophs.

## Material And Methods

### Materials

Phosphate buffered saline (PBS), medium-199, rat ELISA kits for GH, horse serum, penicillin, and streptomycin were purchased from Life Technologies (Grand Island, NY, USA). Fura 2-AM, the secondary Alexa Fluor 568 donkey anti-rabbit antibody, and 4′,6-diamidino-2-phenylindole (DAPI) were from Invitrogen (Eugene, OR). Rodent LH ELISA Kit was from Endocrine Technologies (Newark, CA). The primary antibody and standard for the PRL radioimmunoassay as well as the rabbit anti-PRL antibody for immunocytochemistry were from the National Pituitary Agency and Dr. A. F. Parlow (Harbor-UCLA Medical Center, Torrance, CA). ^125^I-Prolactin was from Perkin Elmer Life Sciences (Boston, MA). Bovine serum albumin (BSA) fraction V and saponin were from MP Biomedicals (Solon, OH). The transcriptor first strand cDNA synthesis kit was from Roche Applied Sciences (Indianapolis, IN), Formaldehyde solution was from Thermo Fisher Scientific (Rockford, IL); Bay K8644, PIK93, UNC3230, and Wm were from Tocris (Bristol, UK). ISA2011B was from Med Chem Express (Monmouth Junction, NJ). TRH and dopamine were from Bachem Americas (Vista, CA). Unless stated otherwise, all other chemicals were obtained from Sigma (St. Louis, MO, USA).

### Animals, Cell Preparation/Culturing and Treatments

**Figure 1:**
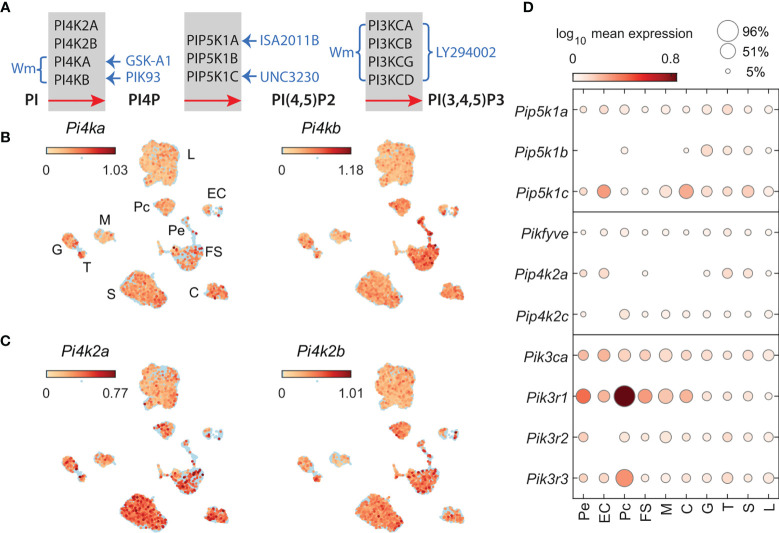
Phosphatidylinositol kinases involved in the generation of PI4P, PI (4,5)P2, and PI (3,4,5)P3 and kinase inhibitors used in this study. **(A)** Inhibition of PIKs by drugs; braces and arrows indicate the enzymes inhibited by inhibitors (blue text). Wortmannin (Wm), a fungal metabolite, is a cell-permeable, irreversible inhibitor of phosphatidylinositol-4-kinases PI4KA and PI4KB in a micromolar concentration range (20) and phosphadidylinositol-3-kinases (PI3Ks) in a nanomolar concentration range (29). PI3KCs are also inhibited by LY294002 in a micromolar concentration range (30). PI4KA is inhibited by GSK-A1 (25) and PI4KB by PIK93 (31), both in a nanomolar concentration range. ISA2011B blocks PIP5K1A (26), whereas UNC3230 inhibits PIP5K1C (27). If not otherwise indicated, we used 10 µM Wm, 100 nM GSK-A1, 100 nM PIK93, 10 µM ISA2011B, 1 µM UNC3230, and 1 and 50 µM LY2394002. Enzymes involved in PI5P production, PIKfyve, a FYVE finger-containing PIK, and PI5P-dependent PI (4,5)P2 production, PIP4K2, phosphatidylinositol-5-phosphate 4-kinases type-2, and their inhibitors, are not shown. **(B, C)** The expression pattern of PIK genes in scRNAseq of freshly dispersed rat pituitary cells. Uniform manifold approximation and projection (UMAP) plots showing the expression of *P4ka* and *Pi4kb* **(B)** and *Pi4k2a* and *Pi4k2b* **(C)** in pituitary cells: L, lactotrophs; G, gonadotrophs; T, thyrotrophs; S, somatotrophs; C, corticotrophs; M, melanotrophs; FS, folliculostellate cells; Pe, pericytes; Pc, pituicytes; and EC, endothelial cells. **(D)**, Cell-type-specific expression of *Pip5k1a-c* (top panel); *Pikfyve, Pip4k2a, Pip4k2c* (middle panel); *Pik3ca, Pik3r1, Pik3r2*, and *Pik3r3* (bottom panel), shown as mean expression level (color code) and percentage of pituitary cell types expressing these genes (area of circles).

Experiments were performed on cultured anterior pituitary cells from normal 75-day-old female Sprague-Dawley rats obtained from Taconic Farms (Germantown, NY). Animals were housed under constant conditions of temperature and humidity, with lights on between 6 AM and 8 PM and water and food ad libitum. Euthanasia was performed by asphyxiation with CO_2_, and the anterior pituitary glands were removed after decapitation. Pituitary tissue was cut into 0.5 x 0.5 mm pieces and treated with trypsin, followed by trypsin inhibitor, and mechanically dispersed as previously described (28). Freshly dispersed pituitary cells were cultured in medium 199 containing Earle’s salts, sodium bicarbonate and supplemented with 10% heat-inactivated horse serum, penicillin (100 units/ml), and streptomycin (100 µg/ml). For immunocytochemistry, 50,000 cells/well were plated on poly-L-lysine-coated eight-well multitest slides (MP Biomedicals, Aurora, OH). For secretory studies, cells were cultured in biocoated 24-well plates (Corning, Kennebunk, ME), 0.25 million per well or in 60 mm petri dishes (Falcon/Becton Dickinson, Franklin lakes, NJ) containing pre-swollen Cytodex-1 beads, 12 million per dish. For RNA extraction, cells were seeded on poly-L-lysine-coated 24-well plates, 1.5 million per well. For calcium experiments, cells were plated at a density of 0.7 million per 25 mm glass coverslip (Thomas Scientific, Logan Township, NJ) coated with poly-D-lysine. In all cases, cells were cultured for approximatively 20 h, washed and treated with several PI4K, PIP5K, and PI3K inhibitors or solvents (**Figure 1A**). All experimental procedures were in accordance with the National Institutes of Health Policy Manual 3040-2: Animal Care and Use in the Intramural Program and were approved by the National Institute of Child Health and Human Development, Animal Care and Use Committee (Animal Protocol 19-041).

### Single-Cell RNA Sequencing Analysis

Dispersed cells from two cell preparations were used for single-cell RNA sequencing (scRNAseq) analysis, as described in Fletcher et al. (32); one preparation was done by dispersion of 30 whole pituitary glands, and the other dispersion was done with 30 separate anterior and intermediate/posterior lobes of the pituitary gland. The whole pituitary sample was run in duplicate, and the anterior and posterior samples were run in separate lanes on a 10X Genomics chromium controller according to manufacturer instructions. The resulting libraries were sequenced on an Illumina HiSeq 2500, and the Cellranger pipeline (10X Genomics) was used for read mapping and transcript counting. A total of 32,455 droplets were recovered from the four lanes. A custom reference genome, cell filtering, differential expression analysis, and ambient RNA removal by SoupX were used as previously described (33). The final data set contained 15,876 cells, for which cell type clusters were identified using known genetic markers: *Lum*, *Col3a1*, and *C7* for pericytes; *Plvap*, *Kdr*, and *Flt1* for endothelial cells; *Lhx2*, *Col25a1*, and *Fgf10* for pituicytes, *Mt2A*, *Aldh3a1*, and *Capn6* for folliculostellate cells; *Pomc* for melanotrophs and corticotrophs, with melanotroph-specific *Oacyl*, *Pax7*, and *Pcsk2*, and corticotroph-specific *Doc2g*, *Avpr1b*, and *Crhr1*; *Lhb*, *Gnrhr*, and *Nr5a1* for gonadotrophs; *Pou1f1* for thyrotroph, somatotrophs and lactotrophs, *Trhr* for thyrotrophs and lactotrophs, with thyrotroph-specific *Tshb* and *Kcnk9*, somatotroph-specific *Gh1*, *Ghrhr*, and *Arhgap36* for somatotrophs, and *Prl*, *Drd2*, and *Agtr1b* for lactotrophs. As expected, the majority of pituicytes and melanotrophs were detected in the posterior pituitary preparation, and pericytes and endothelial cells were present in both the anterior and posterior pituitary preparations. In this study, we use cell-type-specific clustering to examine the expression levels of selected PI3K, PI4K, and PIP5K genes and the percentage of cells of each cell type expressing these genes. Expression of genes of interest was plotted using uniform manifold approximation and projection (UMAP, 34) and percent-expression dot plots using MATLAB (R2018b; The MathWorks, Natick, MA). Single cell RNA sequencing data are accessible at NCBI Gene Expression Omnibus database, accession GSE184319.

### Immunocytochemistry

After overnight incubation, 8-well multitest slides were washed, cells were treated with Wm or 0.1% DMSO for 1, 2, or 3 h, washed twice with PBS, and fixed with cold 4% formaldehyde solution for 10 min. From this point, every step of immunostaining protocol was followed by washing cells three times with PBS. Cells were incubated with rabbit anti-PRL antibody (1:2000), followed by subsequent incubation with secondary antibody (1:1000) for 30 min at room temperature. All antibodies were diluted in PBS containing 0.2% saponin and 0.5% BSA. Cells were mounted with Flouromount-G, with DAPI. All images were acquired on an inverted confocal laser-scanning microscope (LSM 780; Carl Zeiss GmbH, Jena, Germany), using a 63x oil objective. Micrographs were sized, and their brightness and contrast levels adjusted in Fiji image processing software (35). Cells were counted on 8 tile-scan images from 2 independent experiments (3x3).

### qRT-PCR Analysis

Total RNA was extracted from dispersed anterior pituitary cells using RNeasy Plus Mini Kit and reverse transcribed with the Transcriptor First Strand cDNA Synthesis Kit. qRT-PCR was performed in the presence of cDNA (2 ng), TaqMan™ Fast Advanced Master Mix, and TaqMan Gene Expression Assays using the Quantum Studio 6 Flex Real Time PCR System. Target gene expression levels were determined by the comparative 2^-(delta Ct) quantification method using *Gapdh* as the reference gene, which was previously established to be suitable for anterior pituitary tissue (36). Applied Biosystems pre-designed TaqMan Gene Expression Assays were used: prolactin (*Prl*, 00561791_m1) and glyceraldehyde 3-phosphate dehydrogenase gene (*Gapdh*; Rn01462662_g1).

### Single Cell Calcium Measurements

For measurements of intracellular calcium concentration ([Ca^2+^]_i_), cultured pituitary cells were washed and bathed in Krebs-Ringer-like medium containing 2.5 μM Fura 2 AM for 1 h at room temperature. The coverslips were washed in Krebs-Ringer-like medium and mounted on the stage of an inverted Observer-D1 microscope (Carl Zeiss, Oberkochen, Germany) with an attached ORCA-ER camera (Hamamatsu Photonics, Hamamatsu City, Japan) and a Lambda DG-4 wavelength switcher (Sutter, Novato, CA). Hardware control and image analysis were performed using Metafluor software (Molecular Devices, Downingtown, PA). Experiments were performed under a 40X oil-immersion objective during exposure to alternating 340 and 380 nm excitation beams, and the intensity of light emission at 520 nm was followed simultaneously in approximately 20 single cells. Changes in [Ca^2+^]_i_ are represented by the ratio of fluorescence intensities F_340_/F_380_. Single lactotrophs in the mixed population of pituitary cells were identified by their responses to both TRH (100 nM) and dopamine (1 µM), in contrast to thyrotrophs responding only to TRH (100 nM).

### Secretory Studies

Two types of secretory experiments were performed: static incubations and perfused pituitary cells. After overnight incubation, static cultures were washed with medium 199 supplemented with warm HEPES-containing medium-199 supplemented with 0.1% BSA and penicillin and streptomycin to estimate basal PRL release in the presence of phosphatidylinositol kinase (PIK) inhibitors or solvents used as controls. Cells were incubated at 37°C for 1 h, or in time-course studies for 1-3 h; afterwards, medium was removed to measure secreted hormones. To analyze changes in the intracellular content of PRL in these experiments, 0.5 ml of ice-cold 20 mM sodium carbonate buffer was added to wells, and plates were frozen at -80°C. Next, plates were scraped using a 1 ml pipette tip and contents transferred to individual tubes. The process of scraping was repeated with 0.5 ml of fresh buffer or ethanol and added to the tube containing the first extract. Cell extracts were centrifuged at 3,000 rpm to remove cell debris. Samples were stored at -20°C until analysis. In perfusion experiments, both basal and stimulated (25 mM KCl, 1 µM Bay K8644, or 100 nM TRH) release of PRL were studied. After overnight incubation, beads with attached pituitary cells were transferred to 37°C-heated chambers and continuously perfused with warm HEPES-containing medium-199 supplemented with 0.1% BSA and penicillin and streptomycin for 2 h at a flow rate of 0.5 ml/min to establish stable basal secretion. Basal and stimulated PRL release was studied in the presence of PIK inhibitors or solvents, which were continuously applied to cells, and 1- or 3-min fractions of perfused medium were collected and stored at -20°C until hormone measurements. In most experiments, PRL (released and cell content) was measured by radioimmunoassay (RIA) as previously described (37). In some experiments, released PRL, LH, and GH were measured by ELISA, following the manufacturers’ instructions. Released hormones were expressed as ng/ml and cell content of hormones as µg/well.

### Data and Statistical Analysis

Results are presented as representative traces (perfusion experiments) and mean ± SEM values (static incubations and selected perfusion experiments). Statistical analysis was performed by Kruskal-Wallis and Wilcoxon tests, with at least P < 0.01 deemed as statistically significant. Mean and SEM values, statistical analysis, half-maximal inhibitory concentrations (IC_50_) and time to half-maximal inhibition (τ_50_) values were calculated using the KaleidaGraph program (Synergy Software, Reading, PA).

## Results

### Expression of PIK Genes in Anterior Pituitary Cells

To characterize the expression of PIK genes in lactotrophs and other pituitary cell types, we examined the expression levels of these genes using scRNAseq of freshly dispersed anterior and posterior pituitary cells of adult female rats. The UMAP plots shown in **Figure 1** indicate that four PI4K genes, *Pi4ka*, *Pi4kb* (B) *Pi4k2a*, and *Pi4k2b* (C), are expressed in lactotrophs and other hormone-producing cells (somatotrophs, gonadotrophs, thyrotrophs, corticotrophs and melanotrophs) as well as in non-hormonal pituitary cell types (folliculostellate cells, pituicytes, pericytes, and endothelial cells). Among cell types, the percentage of cells expressing these genes varied between 3 and 40, with a mean ± SEM value for all cell types of 18 ± 1.4%. In the lactotroph population, these mRNAs were detected in 14-27% of cells. Among PIP5K1 genes, *Pip5k1c* was expressed at the highest level, followed by *Pip5k1a* and *Pip5k1b* in all cell types, including lactotrophs (**Figure 1D**, top panel). *Pikfyve* and *Pip4k2c* genes, encoding proteins that can support an alternative synthetic pathway of PI(4, 5)P2, are also expressed in lactotrophs and other pituitary cell types (**Figure 1D**, middle panel). Among PI3KC genes, only *Pik3ca* reached the threshold for detection by scRNAseq, whereas all three regulatory subunit genes (*Pik3r1*, *Pik3r2*, and *Pik3r3*) were detected in lactotrophs and other cell types (**Figure 1D**, bottom panel). PI3KCA is tightly associated with a regulatory subunit encoded by the p85alpha gene with three splice forms: p85alpha, p55alpha, and p50alpha (38). However, our scRNAseq system does not discriminate between the various splice forms.

### Wortmannin Inhibits Basal PRL Release

**Figure 2:**
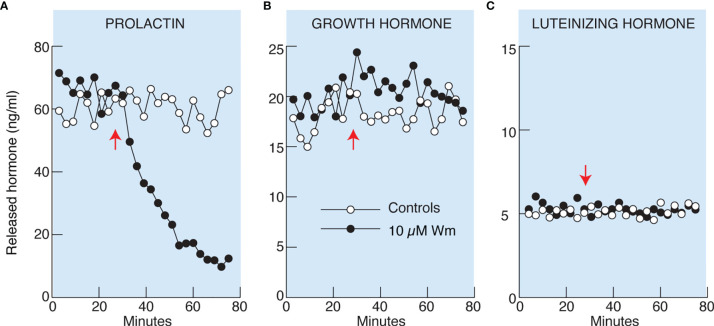
Wortmannin (Wm) inhibited basal prolactin **(A)**, but not growth hormone **(B)** and luteinizing hormone **(C)**, secretion in perfused rat anterior pituitary cells. Freshly dispersed pituitary cells were attached to beads and cultured for 20 hours before the experiments. Perfusion was performed in 0.5 ml volume chambers at a flow rate of 0.5 ml/min and the cells were washed for 2 hours before collecting samples. After the wash period, samples were collected every three minutes for 76 minutes. Vertical arrows indicate the time of application of 10 µM Wm (black circles) or solvent (white circles). Hormone content of the effluent was measured by ELISA. Data shown are representative from three **(A)** and two **(B, C)** similar experiments. For 10 µM Wm-induced inhibition of basal PRL release see also **Figures 4C**, **5B**, **C**, **7C**, **D**, **8B**, and **9D**.

Basal pituitary hormone release comprises regulated exocytosis driven by spontaneous action potential (AP) driven calcium signaling and hormone released by constitutive exocytosis. To study basal hormone release, pituitary cells isolated from adult female rats attached to beads were perfused for 2 h without treatment to wash out previously released hormones, followed by collection of perfusate every 3 min during a 25 min period. This was followed by continuous application of Wm (10 µM in 0.1% DMSO) or solvent (0.1% DMSO) till the end of the experiment (**Figure 2**). Basal PRL, GH, and LH release, measured from the same samples by ELISA, was: 69 ± 3, 18 ± 2, and 5 ± 0.5 ng/ml, respectively. Application of 10 µM Wm caused a rapid decline in PRL release, with an estimated τ_50_ of ~20 min (**Figure 2A**), whereas basal GH (B) and LH (C) release were not affected by this inhibitor.

**Figure 3:**
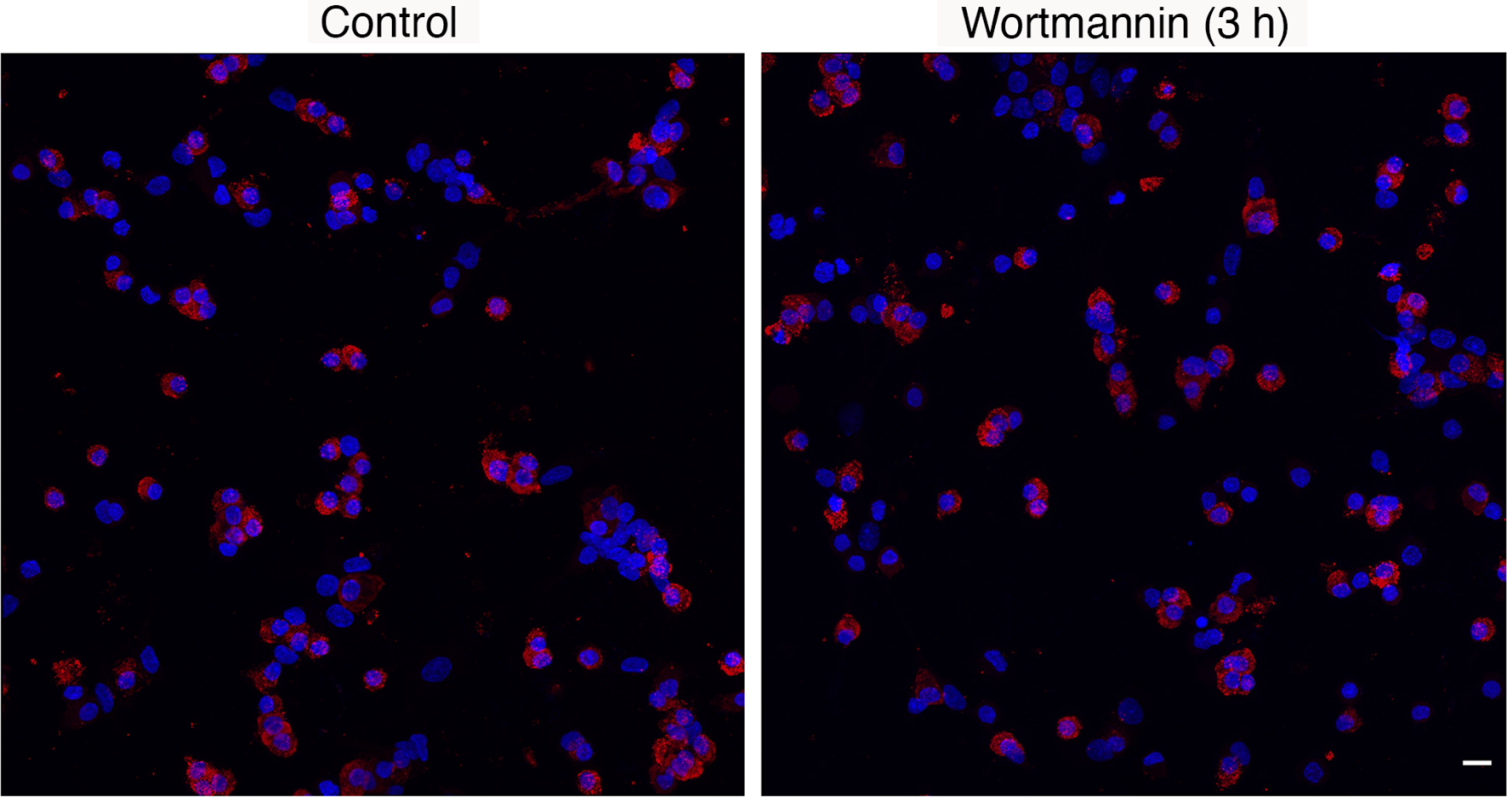
Immunofluorescence analysis of prolactin protein expression in control and Wm (10 µM/3h)-treated pituitary cells. Red indicates prolactin-positive cells; blue indicates cell nuclei stained with DAPI. Scale bar applies to both images: 10 µM. The mean ± SEM values of the percentage of PRL-positive cells in controls was 33.7 ± 2.1 (n = 8) and in Wm-treated cultures was 32.1 ± 2.7 (n = 8).

Immunofluorescence analysis revealed no significant differences in the percentage of lactotrophs during 3 h incubation with 10 µM Wm, when compared to cultures treated with solvent: controls, 1-3 h = 33.7 ± 2.1 (n = 22); Wm, 1 h = 35.7 ± 1.77 (n = 7), Wm, 2 h = 34.9 ± 3.2 (n = 7), and Wm, 3 h = 32.1 ± 2.7 (n = 8). **Figure 3** shows PRL-positive cells in controls and cells treated with Wm for 3 h. Therefore, the cell-type specific action of Wm on basal hormone release in cultured pituitary cells was not caused by selective loss of lactotrophs to Wm but was a genuine effect on PRL secretion.

### PI4KA Mediates the Effect of Wm on Basal PRL Release

**Figure 4:**
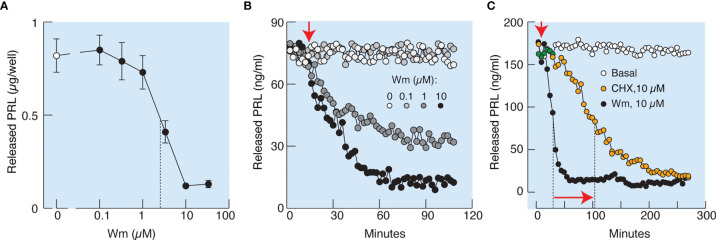
Characterization of Wm-inhibition of basal prolactin secretion in cultured anterior pituitary cells. **(A, B)** Concentration-dependence of Wm effect on prolactin (PRL) release in pituitary cells in static cultures during 3 h incubation **(A)** and in perfused pituitary cells **(B)**. Vertical dotted line in **(A)** indicates the IC_50_ value. In subsequent experiments, Wm was used at 10 µM concentration. **(C)** Comparison of the time-course of effects of Wm (black circles) and cycloheximide (CHX), a protein synthesis inhibitor (orange circles), on basal PRL release. Note the different time scale of this experiment *vs*. panel **(B)**. Vertical dotted lines indicate time points when 50% decrease of hormone secretion was reached, horizontal arrow indicates the difference in half-times, and vertical arrow indicates the beginning of continuous treatments with Wm and CHX. Data points shown in panel A are mean ± SEM of sextuplicate incubation and data shown in panels **(B, C)** are representative from two experiments. In this and following figures, PRL was measured by radioimmunoassay, unless stated otherwise.

**Figure 5:**
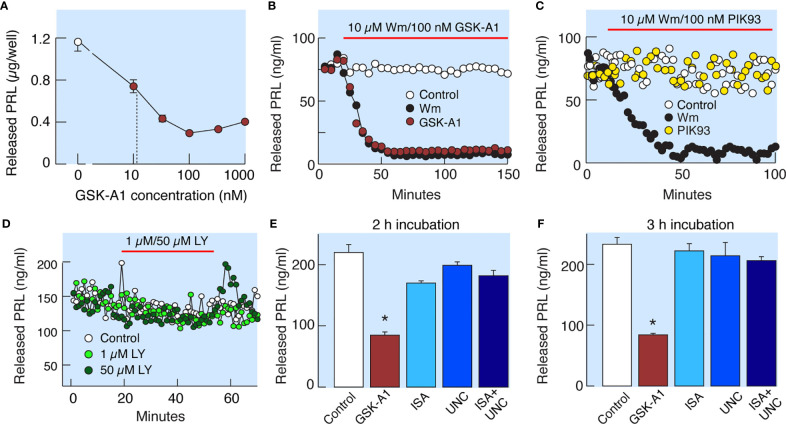
Wm-induced inhibition of PI4KA accounts for inhibition of basal PRL release by Wm in anterior pituitary cells. **(A)** Concentration-dependence of GSK-A1, a PI4KA-sprecific inhibitor, on basal PRL release in static cultures of pituitary cells during 3-h incubation. In subsequent experiments, GSK-A1 was used at a concentration of 100 nM. **(B, C)** In perfused pituitary cells, the inhibitory effect of Wm on PRL release was fully mimicked by application of GSK-A1 **(B)**, but not by PIK93, a PI4KB-specific inhibitor **(C)**. **(D)** Lack of effects of LY294002 (LY), an inhibitor of PI3KC and other PI3Ks, on basal PRL release. In this and following figures, horizontal red bars indicate the duration of treatments with inhibitors and solvents (controls). **(E, F)** Decrease in basal PRL release by addition of PI4KA but not PIP5K1 inhibitors. Cultured cells were incubated with 100 nM GSK-A1, 10 µM ISA2011B (ISA), 1 µM UNC3230 (UNC), and 10 µM ISA2011B plus 1 µM UNC3230 for 2 h **(E)** and 3 h **(F)**. Data points shown in panels **(A, E, F)** are mean ± SEM of sextuplicate incubation; *P < 0.002 *vs* control. The released PRL was measured by RIA **(A–D)** and ELISA **(E, F)**. For time course of GSK-A1 effects on basal PRL release see also **Figure 8B**, left, and **9C**. The lack of effects of PIK93 on basal PRL release is also shown in **Figures 9C, D**.

In further experiments, cultured cells in static incubations were treated with various Wm concentrations for 3 h, and PRL was measured in medium by RIA. **Figure 4A** illustrates that Wm inhibits basal PRL release in the 1 – 10 µM concentration range, with an estimated IC_50_ of ~5.5 µM, and was ineffective in the nanomolar concentration range. In perfused pituitary cells, Wm was ineffective at 0.1 µM but inhibited basal PRL release when administrated at 1 and 10 µM (**Figure 4B**). Therefore, inhibition of basal PRL release induced by Wm at concentration of 10 µM was detected by both methods, ELISA and RIA, confirming the specificity of PRL measurements. The inhibitory Wm action on the basal PRL release was consistently observed, as shown in **Figures 4C**, **5B**, **C**, **7C**, **D**, and **9D**. Basal PRL release in pituitary cells derived from adult male rats was also inhibited by 10 µM Wm (data not shown).

Wm inhibits Class I PI3Ks in the low nanomolar concentration range (19) and PI4Ks in the low to high micromolar concentration range (20). Among the four known PI4Ks, only PI4KA and PI4KB are inhibited by Wm (20). PI4KA is specifically inhibited by GSK-A1 and PI4KB by PIK93 (**Figure 1A**). In static cultures of anterior pituitary cells, GSK-A1 inhibited basal PRL release in a concentration-dependent manner, with an IC_50_ of ~10 nM (**Figure 5A**). In perfused pituitary cells, the rate and level of inhibition of basal PRL release by 100 nM GSK-A1 was highly comparable to the inhibition induced by 10 µM Wm (**Figure 5B**). In contrast, 1 µM PIK93 was not able to replicate the inhibitory action of Wm on basal PRL release (**Figure 5C**). Likewise, the PI3K inhibitor LY294002, did not affect basal PRL release in perfused pituitary cells when applied in 1 and 50 µM for 35 min (**Figure 5D**). Furthermore, ISA2011B (10 µM) and UNC3230 (1 µM), inhibitors of PIP5K1A and PIP5K1C, respectively (26, 27), were ineffective to inhibit basal PRL release when applied alone or together during 2 h (**Figure 5E**) and 3h (**Figure 5F**) incubations. In the same experiments, GSK-A1 significantly (P < 0.01) inhibited PRL release during 2 and 3 h of incubation. Therefore, the inhibitory effects of Wm and GSK-A1 at such concentration ranges suggest a role of PI4KA, but not PI4KB, PI3Ks or PIP5Ks, in basal PRL release, reflecting the effects of the drug on the exocytotic pathway and/or on *de novo* PRL synthesis.

### PI4KA Controls Basal PRL Release Without Affecting *De Novo* PRL Synthesis

**Figure 6:**
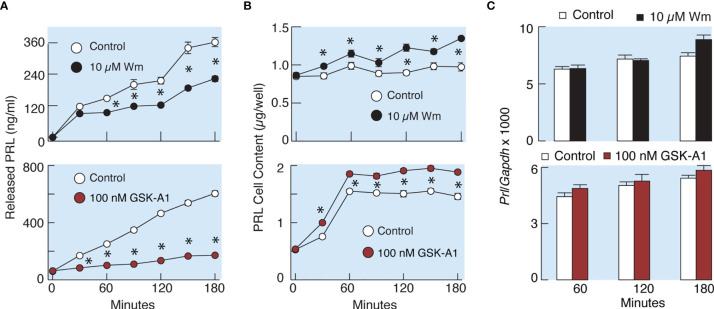
Wm and GSK-A1 induced inhibition of PRL release are not caused by loss of intracellular PRL content and inhibition of PRL gene (*Prl*) expression. **(A)** Time course of Wm- (top) and GSK-A1 (bottom) induced inhibition of basal PRL release. **(B)** Time course of Wm (top) and GSK-A1 (bottom) induced accumulation of intracellular PRL content. **(C)** Lack of effects of Wm (top) and GSK-A1 (bottom) on *Prl* expression in cultured anterior pituitary cells. The experiments were performed in static incubation of cultured anterior pituitary cells: 0.25 million/well **(A, B)** and 1.5. million/well **(C)**. Data points shown are mean ± SEM of sextuplicate incubation; *P < 0.002 between pairs.

To elucidate whether the effects of Wm and GSK-A1 on basal PRL release reflect a direct action on the exocytotic pathway or an indirect action through blockade of *de novo* PRL synthesis, we performed three experiments. In the first experiment, we compared the rates of effects of Wm and cycloheximide, a protein synthesis inhibitor, on basal PRL release in perfused pituitary cells. These experiments, shown in **Figure 4C**, illustrate that the rate of Wm-induced reduction in basal PRL release was faster than the rate of reduction induced by cycloheximide, treatment, suggesting that the exocytotic pathway, rather than PRL synthesis, was blocked by Wm. In the second experiment, we examined the time-course of 10 µM Wm and 100 nM GSK-A1 effects on PRL release (**Figure 6A**) and intracellular PRL content (**Figure 6B**) in static incubation. Both compounds inhibited basal PRL release in a similar time-dependent manner, but the blockade of PRL release was accompanied by increase in intracellular PRL cell content. In third experiment, we examined *Prl* expression (**Figure 6C**) in Wm- (top) and GSK-A1 (bottom) treated cells. Neither treatment significantly affected the expression of this gene during 1, 2, or 3 h incubation. These results indicate that inhibition of PI4KA blocks the exocytotic pathway without affecting *de novo* PRL synthesis.

### PI4KA Controls Stimulated PRL Release Downstream of Calcium Signaling

**Figure 7:**
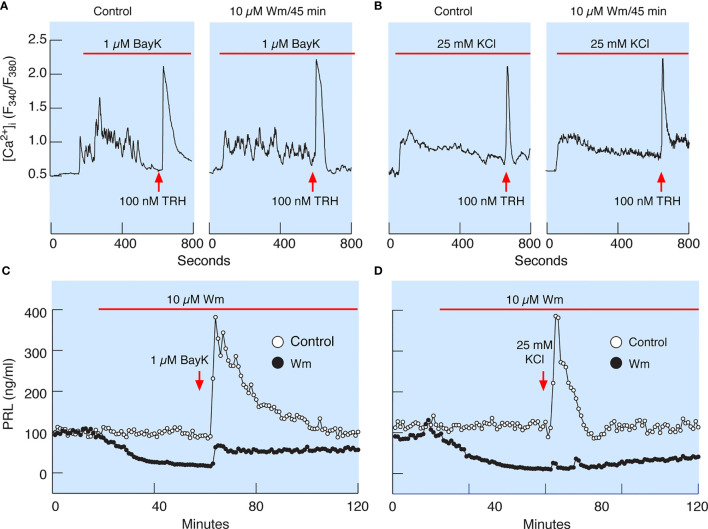
Wm-induced inhibition of PRL release occurs downstream of spontaneous and augmented voltage-gated calcium influx. **(A)** Bay K8644 (BayK), an L-type calcium channel agonist, induced rise in cytosolic calcium concentration ([Ca^2+^]_i_), which was not affected by treatment of cells with 10 µM Wm for 45 min. **(B)** Facilitation of voltage-gated calcium influx by high K^+^-induced depolarization of cells was also not affected by treating the cells with 10 µM Wm for 45 min. Horizontal red bars indicate the duration of BayK and high K^+^ treatments, and the arrows indicate the time of application of thyrotropin-releasing hormone (TRH) in the absence or presence of Wm. In addition to TRH, cells were stimulated with 1 µM dopamine, to identify lactotrophs (not shown). Traces shown are representative from three independent experiments. **(C, D)** Although Wm pretreatment did not affect the facilitated voltage-gated calcium influx, the stimulatory effect of BayK **(C)** and high K^+^ **(D)** on PRL release was dramatically reduced in pituitary cells perfused with Wm-containing medium for 45 minutes. The mean ± SEM values for PRL release (ng/ml) during the first 20 min of BayK treatments in the absence and presence of Wm in four experiments were: 210 ± 13 *vs*. 42 ± 2.4; 146 ± 10 *vs*. 31 ± 1; 184 ± 11 *vs*. 35 ± 2; and 481 ± 24 *vs*. 193 ± 11. The mean ± SEM values for two high potassium experiments in the absence and presence of Wm were 173 ± 21 *vs*. 18 ± 1; and 255 ± 172 *vs*. 5 ± 3. In all cases, P < 0.0001.

In theory, inhibition of PI4KA could cause blockade of spontaneous voltage-gated calcium influx, leading to inhibition of PRL release, or may inhibit the exocytotic pathway downstream of voltage-gated calcium signaling. To clarify this issue, we facilitated voltage-gated calcium influx by the addition of 1 µM Bay K8644, an L-type calcium channel agonist (**Figure 7A**), or by depolarization of the cells by 25 mM KCl (**Figure 7B**) in control cells (left panels) and cells pretreated with 10 µM Wm for 45 min (right panels). In the presence of Bay K8644 or high potassium, 100 nM TRH was added at the end of recording to estimate the potential effects of Wm on calcium mobilizing action of this agonist, which is dependent on the presence of PI (4,5)P_2_ in the plasma membrane (39). These experiments revealed that stimulation of calcium influx was not affected by 45 min application of 10 µM Wm. The experiments further showed no difference in the pattern of TRH-induced calcium mobilization in Wm-treated cells (**Figures 7A, B**). This is an important finding, as it indicates that Wm treatment did not decrease PI (4,5)P2 levels, in agreement with findings in other cell types that Wm and GSK-A1 treatments do not decrease PI (4,5)P2 levels in the absence of phospholipase C activation (40).

Consistent with calcium-secretion coupling in lactotrophs, facilitation of voltage-gated calcium influx by Bay K8644 rapidly increased PRL release in perfused control cells, followed by a prolonged decay in secretion to basal levels (**Figure 7C**). Depolarization of control cells by high potassium also increased PRL release but was followed by a faster return to basal secretion levels (**Figure 7D**). However, in cells treated with 10 µM Wm there was a progressive decay in PRL release, and application of Bay K8644 only slightly recovered PRL release, with a peak in response below the basal levels in controls (**Figure 7C**). High potassium was practically ineffective in elevating PRL release in Wm-treated cells (**Figure 7D**).

**Figure 8:**
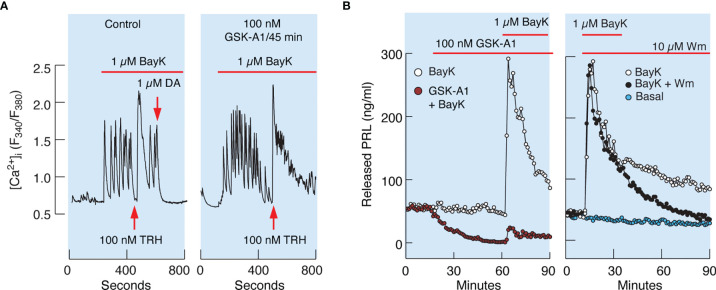
Inhibition of PI4KA by GSK-A1 causes a decrease in BayK-induced PRL release downstream of facilitated voltage-gated calcium influx. **(A)** BayK-stimulated voltage-gated calcium influx (left) and lack of effect of GSK-A1 on the BayK-induced rise in [Ca^2+^]_i_ (right). Time of addition of TRH and Dopamine (DA) is indicated by the arrows. Traces shown are representative from three independent experiments. (**B**, left) In cells treated with GSK-A1, basal PRL release was progressively reduced and BayK was unable to fully restore calcium–secretion coupling. Mean ± SEM values (ng/ml) during 20 min application: BayK 186 ± 12 *vs*. GSK-A1+BayK = 14 ± 1; P < 0.0001. (**B**, right), When Wm and Bay K were applied together, the initial increase in PRL secretion was comparable to that of BayK-induced PRL release; mean ± SEM values (ng/ml) during 20 min application were 182 ± 11 (w/o Wm) *vs*. 176 ± 10 (with Wm). However, in Wm-treated cells, BayK-stimulated PRL release gradually declined, with a rate comparable to that of the inhibition of basal PRL release shown in the left panel, in contrast to control cells.

Similarly, 100 nM GSK-A1 administrated over 45 min did not affect Bay K8644-stimulated voltage-gated calcium influx and TRH-induced calcium mobilization. (**Figure 8A**). TRH receptors are expressed in lactotrophs and thyrotrophs, but only lactotrophs respond to application of dopamine administration (**Figure 8A**, left). In contrast to calcium signaling, GSK-A1 dramatically reduced Bay K8644-stimulated PRL release in perfused pituitary cells (**Figure 8B**, left). When Wm and Bay K8644 were added at the same time, however, no inhibition of the early spike of PRL release was observed (**Figure 8B**, right), suggesting that blockade of calcium-secretion coupling takes time to develop, likely because it takes time for PI4P levels to decrease after blockade of PI4KA (41).

**Figure 9:**
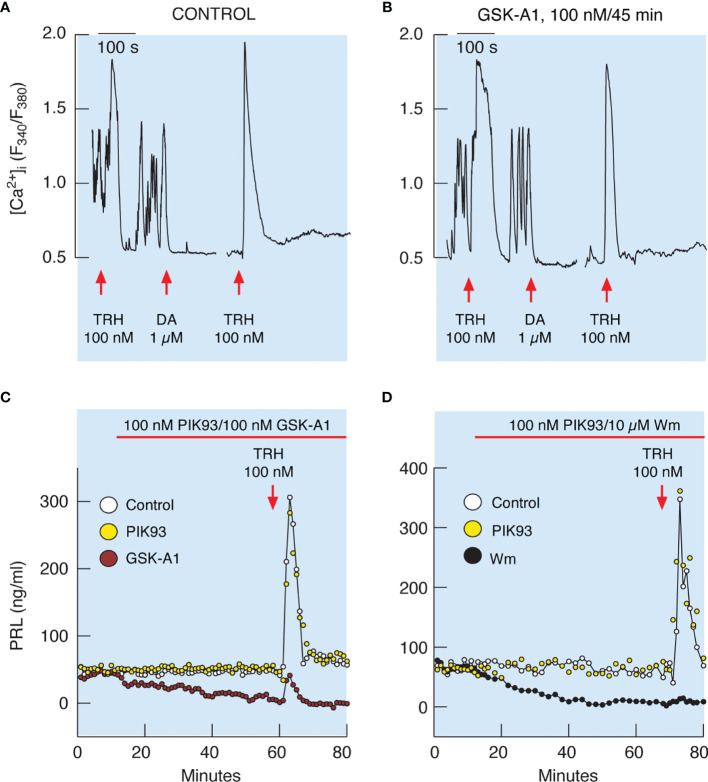
Inhibition of PI4KA blocks TRH-stimulated PRL release downstream of TRH-induced calcium mobilization from intracellular pools. **(A, B)** To identify single lactotrophs, control **(A)** and GSK-A1-pretreated **(B)** cells were treated with TRH and dopamine (DA), washed for 15 min, and restimulated with TRH. GSK-A1 was added 45 min before recording, during TRH and DA treatments, and during the rinsing periods. DA blocks voltage-gated calcium influx, which is also a useful pharmacological tool for demonstrating extracellular calcium independence of spike response and extracellular calcium dependence of sustained calcium transients, as shown by another application of TRH in the presence of dopamine. Traces shown are representative from three independent experiments. **(C, D)** TRH-induced PRL release was dramatically reduced in GSK-A1- and Wm-treated cells, but not in PIK93-treated cells. Mean ± SEM values (ng/ml) during 20 min TRH application were: **(C)** TRH 107 ± 14 ng/ml, GSK-A1+ TRH = 14 ± 3 (P < 0.0001), and PIK+TRH 107 ± 15; **(D)** TRH 172 ± 31 ng/ml, Wm + TRH = 10 ± 1 (P < 0.0001), and PIK93+TRH 205 ± 30.

It has been well established that TRH-stimulated calcium mobilization from the intracellular pool increases the release of PRL *in vitro* (18). As mentioned earlier, TRH-stimulated calcium mobilization was not affected by prolonged (45 min) treatment with 10 µM Wm and 100 nM GSK-A1 (**Figures 7** and **8**). In general, TRH-induced calcium signaling consist of an early spike response, which reflects calcium mobilization from intracellular pools, and sustained calcium transients, reflecting extracellular calcium influx (18, 39). Both calcium signaling phases were unaffected in cells treated with GSK-A1 for 45 min (**Figures 9** **A, B**). Dopamine abolished sustained intracellular calcium transients, confirming that they were driven by voltage-gated calcium channels and that the selected cells were lactotrophs (**Figures 9A, B**). Additional administration of TRH in the presence of dopamine shows the independence of the spike phase from calcium influx in the controls (**Figure 9A**) and that the blockade of PI4KA does not affect the repeated mobilization of calcium from intracellular stores (**Figure 9B**).

**Figure 9C** shows that the profile of TRH-induced PRL release in perfused control cells is biphasic, composed of an early spike phase and a sustained plateau phase, and is therefore comparable to the TRH-stimulated calcium signaling profile. In contrast to calcium signaling, however, TRH-induced PRL release was dramatically reduced in GSK-A1 (**Figure 9C**) and Wm (**Figure 9D**) treated cells. Administration of 1 µM PIK93, a PI4KB inhibitor, was ineffective (**Figures 9C, D**). Therefore, functional PI4KA is also critical for the release of PRL induced by calcium mobilization.

## Discussion

In this study, we examined the expression of the PIK genes that control production of phosphoinositides, including PI4P, PI (4,5)P2, and PI (3,4,5)P3, and the possible role of these signaling molecules in hormone secretion in pituitary cells. The focus of this study was on PRL-secreting lactotrophs of the anterior pituitary gland. The data indicate quantitative rather than qualitative differences in PIK gene expression among pituitary cell types. Lactotrophs and all other cell types expressed all four PI4K genes and three PIP5K1 genes, as well as *Pikfyve* and *Pip4k2c*, indicating that the pituitary gland is well equipped for PI4P and PI (4,5)P2 synthesis. Among the four members of class I PI3KC genes, *Pik3ca* expression was detected at the single cell level as well as *Pik3r1*, which encodes a regulatory subunit of this enzyme. However, the presence of the PI3K regulatory subunit genes *Pik3r2* and *Pik3r3* in pituitary cells is indicative that other forms of PI3KC could also be expressed in pituitary cells but below the threshold of detection by scRNAseq. The expression of these genes was also observed in scRNAseq analysis of adult male pituitary cells (33); the NCBI Gene Expression Omnibus, GSE:132224.

We performed a pharmacological assessment of the role of these enzymes in hormone secretion initially using Wm, which effectively blocks all PI3Ks (19) and PI4KA/PI4KB (42). These experiments showed that Wm blocks basal PRL release in cultured pituitary cells from females in a time- and concentration-dependent manner, without affecting basal LH and GH secretion. We also observed inhibitory effect of Wm on PRL release in pituitary cells from adult male rats. Therefore, although lactotrophs, somatotrophs, and gonadotrophs all express the Wm-sensitive PIK gene products at comparable levels, only PRL secretion is affected. To elucidate the mechanism by which Wm inhibits PRL release, but not LH and GH release, and which kinases account for inhibitory action, we pursued several leads in this study.

In general, progressive inhibition of PRL release by Wm during continuous administration could reflect inhibition of *de novo* PRL synthesis, leading to a gradual depletion of the secretory pool and a decrease in PRL release. However, three lines of evidence challenge this hypothesis. First, the τ_50_ of Wm-induced inhibition of PRL release was shorter than that induced by cycloheximide, a protein synthesis blocker. Second, Wm did not affect the expression of the PRL gene. Third, Wm blockade of PRL release was not accompanied by a decrease in the intracellular PRL pool, but rather by its significant increase. Therefore, Wm does not affect prolactin synthesis, but inhibits the release of stored hormone in secretory vesicles of pituitary lactotrophs.

It has been well established that in lactotroph cells, spontaneous plateau-bursting type APs, accompanied by [Ca^2+^]_i_ transients, are of sufficient amplitude to trigger calcium-dependent hormone release that contributes to what is called basal secretion (16). It has also been well established that blocking spontaneous electrical activity by removal of bath calcium or adding dopamine abolishes firing of APs, calcium signaling, and basal PRL release (16, 17). Conversely, high potassium and Bay K8644 stimulation of PRL release, also reported by others (43–45), confirms the critical role of voltage-gated calcium influx in the hormone release process. Therefore, it was reasonable to hypothesize that Wm inhibits PRL release by blocking the spontaneous and/or the facilitated voltage-gated calcium influx. However, we found that Wm treatment did not affect voltage-gated calcium influx. Furthermore, we showed that Wm inhibited TRH-stimulated PRL release without affecting IP3-dependent calcium mobilization. Together, these observations indicate that Wm inhibits basal as well as stimulated PRL secretion downstream of the calcium generating molecular events.

Wm is not a specific inhibitor of PIK enzymes, as it inhibits several lipid (and protein) kinases with different potencies. Class I PI3Ks are inhibited by Wm with an IC_50_ of 1-5 nM (29); class II PI3K with an IC_50_ of 1.2 nM (7); and class III PI3Ks, with an IC_50_ of 50-300 nM (46). Wm also inhibits the PI3K-related kinases with different potencies (low to high IC_50_s): PRKDC, 16 nM; SMG1, 60 nM; ATM, 100-150 nM; mTOR, 200 nM; and ATR, 1.8 µM (47–50). The polo-like kinase is also inhibited by Wm with an IC_50_ of 24-48 nM (51) as well as smooth muscle myosin light chain kinase with an IC_50_ of 0.17 µM (52), mitogen-activated protein kinases, with an IC_50_ of 0.2-0.3 µM (53), and PI4Ks, with an IC_50_ of 0.3 µM (20). Our scRNAseq data indicate that *Atr*, *Plk1*, and *Mylk* are not expressed in pituitary cells, but the other genes in this list are present.

Due to the documented role of PI3Ks in fish pituitary hormone secretion (14, 54, 55) and their inhibition by Wm, we initially hypothesized that Wm blockade of PI3KCs in lactotrophs caused inhibition of PRL release. We tested this hypothesis in two ways. Our concentration-dependence study with pituitary cells in static incubations and perfused cells showed no effects of Wm in the nanomolar range of concentration, which are sufficient to block PI3KCs. Administration of LY294002, another established PI3KC inhibitor (21), also did not affect basal PRL release. Therefore, inhibition of PI3KCs by these compounds does not account for inhibition of spontaneous and stimulated PRL release during 3 h of treatment.

Because ATR and smooth muscle myosin light chain kinase are not detectable in lactotrophs, and high concentrations of Wm also inhibit type-III PI4Ks, we focused on the latter enzymes. The inhibitory action of Wm on PRL release was mimicked by GSK-A1, a highly potent and specific PI4KA inhibitor, with an IC_50_ of ~10 nM (25). The time-courses of PRL release inhibition induced by Wm and GSK-A1 were also identical. Furthermore, GSK-A1-induced inhibition of PRL release was accompanied by an increase in cellular PRL content. Finally, GSK-A1 inhibited PRL release without affecting Bay K8644-, high potassium-, and TRH-induced calcium signaling. In contrast, PIK93, which inhibits PI4KB *in vitro* with an IC_50_ of 14 nM (56), did not inhibit basal and TRH-stimulated PRL release when administrated in up to 1 µM concentration. These observations strongly support a role of PI4KA in calcium dependent PRL release.

Since PI4P is a substrate for PI(4, 5)P2 production, there was a possibility that PI(4, 5)P2 changes account for the inhibition of PRL release by blocking PI4KA. In general, the role of PI(4, 5)P2 in the control of ion channels is well established, including voltage-gated potassium channels (10, 57), inwardly rectifying potassium channels (58, 59), calcium-activated potassium channels (60, 61), and voltage-gated calcium channels (62). PI(4, 5)P2 also enhances calcium-secretion coupling downstream of calcium signaling (11, 12). Although the genes for these channels are also expressed in pituitary lactotrophs (33), the lack of effect of Wm and GSK-A1 on voltage-gated and TRH-stimulated calcium signaling suggests that PI(4, 5)P2 production/metabolism is not significantly affected by PI4KA inhibition on the timescale of the current studies.

As stated above, PI(4, 5)P2 can be synthesized *via* two enzymatic pathways: the phosphorylation of PI4P at position 5 of the inositol head group by PIP5Ks or phosphorylation of PI5P at position 4 by PIP4Ks (9). However, the bulk of the PI(4, 5)P2 in animal cells is synthesized by the PI4K-PIP5K pathway (63). In experiments using the PIP5K1A and PIP5K1C inhibitors, ISA2011B and UNC3230, respectively, no inhibition of PRL release was observed. Assuming that these inhibitors indeed inhibited PI(4, 5)P2 synthesis, these data suggested that a decrease in basal PI(4, 5)P2 is not responsible for the Wm-inhibition of PRL release. This was in agreement with previous studies showing that Wm or GSK-A1 treatment does not decrease PI(4, 5)P2 levels in spite of their strong effects on PI4P levels (20, 25, 64). It is therefore reasonable to conclude that plasma membrane PI4P is critical for basal and stimulated PRL release independently of PI(4, 5)P2.

In conclusion, we present here pharmacological evidence for the critical role of PI4KA in the release of PRL in mammalian lactotrophs, highlighting the contribution of plasma membrane PI4P in supporting calcium secretion coupling downstream of calcium signaling. Others also observed that the addition of PI4P potentiates calcium-induced insulin exocytosis (65). The role of PI4P in vesicle exit from the Golgi is well established, but PI4KB plays a major role in this process (66). PI4P was also reported to regulate exocytosis in *S. cerevisiae* by promoting vesicle docking (67). Additional studies are needed to elucidate the exact molecular steps controlled by PI4P in the exocytotic pathway in mammalian lactotrophs downstream of calcium signaling. The existence of conditional knockout PI4KA mice (25, 64), also provides an opportunity to test this hypothesis more directly by silencing this gene in different hormone-producing cells.

## Data Availability Statement

The raw data supporting the conclusions of this article will be made available by the authors, without undue reservation. Single cell RNA sequencing data are accessible at NCBI GEO database, accession GSE184319.

## Ethics Statement

The animal study was reviewed and approved by The National Institute of Child Health and Human Development, Animal Care and Use Committee (Animal Protocol 19-041).

## Author Contributions

Conceptualization: SS and TB. Experimental Work: MK, AG-I, MT, RP, KS and SJS. Writing – original draft: SS. Writing – review and editing: all authors. Data analysis and figure preparation: SS and PF. Supervision and funding acquisition: SS, AS, and TB. All authors contributed to the article and approved the submitted version.

## Funding

This work was supported by National Institutes of Health grants from the Intramural Research Program of the Eunice Kennedy Shriver NICHD and NIDDK.

## Conflict of Interest

The authors declare that the research was conducted in the absence of any commercial or financial relationships that could be construed as a potential conflict of interest.

## Publisher’s Note

All claims expressed in this article are solely those of the authors and do not necessarily represent those of their affiliated organizations, or those of the publisher, the editors and the reviewers. Any product that may be evaluated in this article, or claim that may be made by its manufacturer, is not guaranteed or endorsed by the publisher.
